# Exploring Risk Perception and Attitudes to Miscarriage and Congenital Anomaly in Rural Western Kenya

**DOI:** 10.1371/journal.pone.0080551

**Published:** 2013-11-13

**Authors:** Stephanie Dellicour, Meghna Desai, Linda Mason, Beatrice Odidi, George Aol, Penelope A. Phillips-Howard, Kayla F. Laserson, Feiko O. ter Kuile

**Affiliations:** 1 Malaria Branch, Kenya Medical Research Institute (KEMRI)/ Centers for Disease Control and Prevention (CDC) Research and Public Health Collaboration, Kisumu, Nyanza Province, Kenya; 2 Department of Clinical Sciences, Liverpool School of Tropical Medicine, Liverpool, Merseyside, United Kingdom; 3 Center for Global Health, Centers for Disease Control and Prevention, Atlanta, Georgia, United States of America; 4 International Emerging Infection Branch, Kenya Medical Research Institute/ Centers for Disease Control and Prevention Research and Public Health Collaboration, Kisumu, Nyanza Province, Kenya Research and Public Health Collaboration, Kisumu, Nyanza Province, Kenya; 5 Health and Demographic Surveillance System Branch, Kenya Medical Research Institute/Centers for Disease Control and Prevention Research and Public Health Collaboration, Kisumu, Nyanza Province, Kenya; Tabriz University of Medical Sciences, Iran (Islamic Republic Of)

## Abstract

**Background:**

Understanding the socio-cultural context and perceptions of adverse pregnancy outcomes is important for informing the best approaches for public health programs. This article describes the perceptions, beliefs and health-seeking behaviours of women from rural western Kenya regarding congenital anomalies and miscarriages.

**Methods:**

Ten focus group discussions (FGDs) were undertaken in a rural district in western Kenya in September 2010. The FGDs included separate groups consisting of adult women of childbearing age, adolescent girls, recently pregnant women, traditional birth attendants and mothers of children with a birth defect. Participants were selected purposively. A deductive thematic framework approach using the questions from the FGD guides was used to analyse the transcripts.

**Results:**

There was substantial overlap between perceived causes of miscarriages and congenital anomalies and these were broadly categorized into two groups: biomedical and cultural. The biomedical causes included medications, illnesses, physical and emotional stresses, as well as hereditary causes. Cultural beliefs mostly related to the breaking of a taboo or not following cultural norms. Mothers were often stigmatised and blamed following miscarriage, or the birth of a child with a congenital anomaly. Often, women did not seek care following miscarriage unless there was a complication. Most reported that children with a congenital anomaly were neglected either because of lack of knowledge of where care could be sought or because these children brought shame to the family and were hidden from society.

**Conclusion:**

The local explanatory model of miscarriage and congenital anomalies covered many perceived causes within biomedical and cultural beliefs. Some of these fuelled stigmatisation and blame of the mother. Understanding of these beliefs, improving access to information about the possible causes of adverse outcomes, and greater collaboration between traditional healers and healthcare providers may help to reduce stigma and increase access to formal healthcare providers.

## Introduction

In Kenya, as in most countries of sub-Saharan Africa, there is a lack of information on the risk of adverse pregnancy outcomes. Reviews using regional estimates project the risk of miscarriages and stillbirths in 2007 to be 12.2% and 3.3% per pregnancy, respectively [1,2]. Appropriate and prompt management of adverse pregnancy outcomes can reduce maternal mortality. For example, the two-fold higher risk of maternal death following miscarriage compared to those who had a live-birth [3,4] mainly reflects poor management of retained products and subsequent infection. 

Globally, it is estimated that every year over three million children die due to congenital anomalies (defined as structural or functional anomalies which occur during the embryo-fetal development and are present at the time of birth). In addition, another three million babies born with a congenital anomaly and do not access care at birth may be disabled for life [5]. The risk of major congenital anomalies in industrialised countries is around 3-5% [6,7]. It is unclear if this is similar in low income countries or if it is higher due to a variety of factors such as different health-seeking behaviours, poor nutrition, a higher prevalence of infectious diseases, weak health systems and a higher availability of a wide range of prescription-only drugs over-the-counter [5,8,9]. This availability of drugs, and the high prevalence of infectious disease, means that pregnant women are more likely to be exposed to teratogenic drugs or drugs with limited information on their safety for pregnant patients. Further, misuse of drugs for induced abortion also has political ramifications, particularly in countries with strict abortion laws and limited access to contraceptive and family planning services. Due to limited information and knowledge, infants born with a congenital anomaly might miss the opportunity to have access to appropriate care in time and suffer the social and economic burden of lifelong disabilities. Receipt of appropriate care significantly impacts the prognosis of newborns with a congenital malformation; for children born with club foot, for example, this can be the difference between a life with limited mobility (and often loss of social and economic opportunities) and that of normal active life with painless functional feet. 

Understanding the local perception of health, stigma, and disclosure is essential to improve access to health services and for patient uptake of new programmes [10,11]. However, women’s perceptions, clients and service providers understanding and attitudes towards adverse pregnancy outcomes, and how these affect health-seeking behaviour, have received scant research attention. This paper describes the results from a qualitative study that explored the perceived causes of miscarriages and congenital anomalies, stigma associated with these adverse outcomes, and issues around disclosure and health-seeking behaviour. 

## Methods

This study was part of a formative research program carried out to inform the best approach to setting up a prospective pharmacovigilance pregnancy cohort to monitor the use of medications during pregnancy, the safety of these drugs in pregnancy, and their impact on birth outcomes. There was no teratogenic drug monitoring program at the time we conducted focus group discussions in the study area. Topics around the socio-cultural context of pregnancy and adverse pregnancy outcomes were explored. 

### Study site

The study took place in Rarieda District, Nyanza Province, in western Kenya (within Siaya County since 2013). This area has been under continuous surveillance as part of the Kenya Medical Research Institute and the US Centers for Disease Control and Prevention (KEMRI/CDC) Health and Demographic Surveillance System (HDSS) since 2001 [12]. Subsistence farming is the main occupation in this area and more than 95% of the population are from the Luo ethnic group [13]. The prevalence of malaria (KEMRI/CDC, unpublished data), HIV [14] and TB [15] are high. Malaria transmission is intense and holo-endemic with peaks after the two rainy seasons usually in June-July and November-December. HIV prevalence in Nyanza province is 13.9% (16.0% among women and 11.4% among men) compared to a national prevalence of 6.3% [14]. HIV prevalence among pregnant women is around 27% [16]. The total fertility rate in this area calculated for 2009 was 4.3 live births per woman [17]. Only 17% of females have had a secondary school education, while 19% have neither primary nor secondary school education [17]. 

### Participants

Females, aged 15 years and older, purposively sampled by village based KEMRI/CDC fieldworkers (village reporters; VRs) were invited to be part of the focus group discussions (FGDs). VRs are community members involved with sensitisation activities for new initiatives or research projects in their villages. Prior to participant selection, VRs were trained on the aims of the formative research and the characteristics of the participants required for each FGD by the study moderator (B.O.), a Kenyan social scientist and the note-taker. The different FGD groups consisted of adult women of childbearing age (WOCBAs; 18-49 years), adolescent girls (15-18 years), recently or currently pregnant women, traditional birth attendants (TBAs; locally called *Nyamrerwas*) and one group of mothers of children born with a congenital anomaly. Each group consisted of no more than one woman from each village. The separate groups were selected to have fairly homogenous demographic characteristics and avoided dominance from older women, enabling freer discussions. A total of 10 focus groups (n=90 participants) were conducted between September 1st and September 22nd, 2010. 

### Study procedures

The FGDs took place in central locations such as schools, or churches, that were easily accessible to all participants, and where privacy could be maintained. Information on the pharmacovigilance study and the formative research was discussed at community meetings with village chiefs and counsellors as well as with a community advisory board (CAB, comprising stakeholders and members of the community who provide advice to researchers on proposed studies). Individual participants gave consent after the information sheet had been verbally explained and discussed. FGD guides were semi-structured, open-ended and probing. The discussion guides were initially written in English and then translated to Dholuo, the local dialect, by the Kenyan social scientist and moderator. The FGD guides covered topics relating to pregnancy recognition, disclosure, health-seeking behaviour and pregnancy related behaviour change as well as use of medication and herbal remedies during pregnancy, practices around delivery, perception of adverse outcomes and unwanted pregnancies. Not all topics were covered by all groups due to time limitations. The FGDs ranged from one to two hours. The moderator and note-taker, both females from the study area, had many years of fieldwork experience, including FGDs, and were trained on the study protocol and tools before the first FGD. All FGDs were recorded using audiotapes. Audio files were transcribed verbatim and translated by the moderator and two independent transcribers; this activity was guided by the notes taken during the FGDs. Each transcript was reviewed by the moderator to ensure consistency and accuracy was maintained.

### Data analysis

A deductive thematic framework approach was used to provide a detailed account of a group of themes using the questions from the FGD guides on perception, attitudes, and risk factors associated with adverse pregnancy outcomes within the data. Each transcript was uploaded to QSR Nvivo 9 software (QSR International Pty Ltd, Melbourne, Australia). The code structure was developed according to the topic guide and the codes were applied to each transcript in a systematic fashion across the entire dataset by collating data relevant to each code. Additional codes were identified inductively from the transcripts. The coding was done independently by S.D. and L.M. and comparisons made; any areas of disagreement were discussed and resolved on revisiting the data. Thematic maps were generated to visualise the main themes and sub-themes and how they connected. Tree maps were generated using Nvivo to compare coding references between the different sub-groups. Table S1 provides a list of the main themes and sub-themes.

### Ethics

The protocol and consent procedures were reviewed and approved by 1) the Kenya Medical Research Institute (KEMRI, Nairobi, Kenya) National Ethics Review Committee; 2) the US Centers for Disease Control and Prevention (CDC, Atlanta, GA, USA) IRB and 3)the Liverpool School of Tropical Medicine (LSTM, UK) Research Ethics Committee. Informed consent was obtained verbally in the local dialect (Dholuo) and tape recorded. This informed consent procedure was approved by all 3 ethics committees. This is common procedure for FGDs as written consent can sometime make participant wary or guarded, particularly in a setting were illiteracy is common and would require finding an appropriate witness.

## Results

Table 1 provides a summary of the participants’ characteristics. The findings are presented as three main themes relating to the specific research questions: 1) Perceived causes of miscarriages and congenital anomalies, 2) stigma and the community perception of miscarriages and congenital anomalies, and 3) health-seeking behaviours for miscarriages and congenital anomalies. There was no major difference observed in the beliefs and themes that emerged between the different groups (i.e. WOCBAs compared with adolescents or TBAs or mothers of children born with a congenital anomaly) and the findings are consequently presented jointly for all groups.

**Table 1:** Summary of Focus Group Discussions Participant Characteristics.

**FGD#**	**Group**	**N**	**Average Age in years**	**Average Gravidity (range)**	**Marital status**			**Education**		
					**Single**	**Married**	**Widowed**	**None**	**Primary**	**Secondary**
FGD1	WOCBA	10	38	7 (3-10)	0	9 (90%)	1 (10%)	0	7 (70%)	3 (30%)
FGD2	WOCBA	9	31	5 (1-9)	0	9(100%)	0	0	8 (89%)	1 (11%)
FGD3	Recently Pregnant	8	32	6 (2-10)	0	7 (88%)	1 (13%)	0	8 (100%)	0
FGD4	TBA/Nyamrerwas	10	54	7 (4-12)	0	4 (40%)	6 (60%)	3 (30%)	2 (20%)	5 (50%)
FGD5	Adolescent	9	16	0	9 (100%)	0	0	0	9 (100%)	0
FGD6	Adolescent	9	17	0	9 (100%)	0	0	0	9 (100%)	0
FGD7	Recently Pregnant	9	28	4 (2-7)	0	9 (100%)	0	0	8 (89%)	1 (11%)
FGD8	TBA/Nyamrerwas	8	41	6 (2-10)	0	8 (100%)	0	1 (13%)	7 (88%)	0
FGD9	WOCBA	9	31	5 (1-10)	1 (11%)	6 (67%)	2 (22%)	1 (11%)	6 (67%)	2 (22%)
FGD10	Mothers of children with congenital anomalies	9	31	5 (1-10)	2 (22%)	6 (67%)	1 (11%)	1 (11%)	8 (89%)	0
**Overall**		**90**	**32**	**5 (0-12)**	**21 (23%)**	**58 (64%)**	**11 (12%)**	**6 (7%)**	**72 (80%)**	**12 (13%)**

Abbreviations: WOCBA- women of childbearing age; TBA- traditional birth attendant

No definition of congenital anomaly was provided to the participants but a wide range of anomalies, both structural and functional, were cited when participants were asked about the type of congenital anomalies they had observed in the community. The most frequently mentioned anomaly was deformity of the hand, followed by mental retardation, and paralysis. Deafness, cleft-lip and eye anomalies were also mentioned by different groups. Explicit anomalies were not specified during the discussion on causes, stigma and health seeking behaviours; therefore we refer to congenital anomalies in general in the text.

### Perception of causes of miscarriages and congenital anomalies

Many different possible causes of miscarriages and congenital anomalies were reported with a significant overlap between these two outcomes. We divided these into two broad groups: those with a biomedical basis and those with a cultural basis.

#### 1: Biomedical Causes

The role and potential dangers of biomedical causes were thematically sub-grouped into medications, illness, physical and emotional stress, and hereditary causes. 

Most women reported that certain drugs could lead to miscarriages or congenital anomalies, but only if they were not prescribed, or if the dose taken was above that prescribed by a healthcare worker. There was an overarching theme of trust by the women that any treatment given or prescribed by clinicians would be safe. One participant mentioned that a pregnant woman should only take medicines if the illness is confirmed. This infers a natural understanding of the concept of balancing the risks and benefits of treatment (i.e. it is only worth taking the risk of consuming medicines if one is truly ill) by this participant. When asked about reservations over the ingestion of drugs in pregnancy, two separate groups brought up the issue of nausea and heightened sensitivity to smell (i.e. relating this to difficultly in taking drugs orally during pregnancy). Thus, it was suggested that pregnant women should be given injections rather than tablets. This implies that reluctance to take medication in pregnancy is associated with pregnancy related nausea, rather than a fear of an adverse effect on the pregnancy or the fetus. Only one participant mentioned fear of side-effects (itching and drowsiness for the pregnant women) as a reason for not taking the drugs given to her during antenatal care.

“I’m requesting that if one is pregnant you should avoid the drugs, it should just be injection given when one is sick. They don’t take drugs. The drugs that I was given in 1997 when I gave birth just expired the other day. So it should be the injection which you come and get.” (*FGD2, P9*)

“When some women go to the clinic and are given medicine because when you go to the clinic you are given medicine for blood, you are given medicine that would give energy…when they are given such medicine, they do not take saying that it smells bad.” (*FGD1, P6*)

“A pregnant woman should not just take medicine if it is not prescribed by the doctor.” (*FGD3, P1*)

Family planning drugs were considered to be distinct types of drugs, separate from medications used for illness, and all groups believed that these could lead to congenital anomalies.

“You find that when you give birth to a child with a defect then people would say it is because of the family planning that has caused it.” (*FGD10, P2*)

Other drugs also deemed to be potentially harmful for the pregnancy were antimalarials, including chloroquine (which is no longer recommended for treatment due to malaria parasite resistance ), quinine (which is considered safe for use in all trimesters of pregnancy and used for severe and complicated malaria which in itself can cause miscarriage), and artemether-lumefantrine (which is the first line treatment for malaria but not recommended for use in the first trimester of pregnancy due to its unknown safety). Chloroquine and quinine were drugs reported to be used by pregnant women within the community to induce abortions for unwanted pregnancies. Anti-retroviral drugs were mentioned by one participant as having the potential to cause congenital anomalies. One participant noted antibiotics could hinder bone development of the fetus.

“[M: Which other way would you use to do abortion?]In some cases I hear people overdose. [What overdose?] For the tablets, there are some drugs if they take like the malariaquine [chloroquine] if you overdose then you will abort.” (*FGD7, p5*)

When asked about the potential risk period for taking medication during pregnancy only one participant mentioned that drugs should be avoided during the first three months of pregnancy. The majority mentioned drugs should be avoided closer to the time of delivery.

Disease or infections were mentioned by all groups as a potential cause of miscarriage. Most participants simply cited ‘disease’ without being specific. However, malaria was mentioned by a few participants, and one person cited HIV. Other illnesses, particularly sexually transmitted infections (gonorrhoea was specifically mentioned), and measles, were commonly cited causes of congenital anomalies.

“Like gonorrhoea maybe you were sick and you don’t seek treatment, so if you gave birth to a child he/she must be disabled in some way.” (*FGD9, P1*)

Participants considered that adverse pregnancy outcomes were the consequence of pregnant women not attending antenatal care services, or not completing their vaccinations (supposedly tetanus vaccine, which is provided routinely through ANC). This could be interpreted as a perception that pregnant women are vulnerable to adverse effects from an illness, which would have been prevented, had the woman attended ANC services. 

Carrying out strenuous work while pregnant was mentioned by all groups as a potential cause of miscarriage. A few respondents also thought physical trauma (such as trauma near or around the uterus) could lead to miscarriage. Women also considered that adverse effects were more likely among women who had conceived many times, suggesting women understood the physical toll of many pregnancies and short birth intervals between pregnancies. Similarly, the position of the fetus in the uterus was reported by one TBA as a potential cause of deformity. Emotional distress such as being shocked by bad news or arguing with a husband/partner was considered to be dangerous for the pregnancy, and could lead to pregnancy loss. Being raped was also mentioned as a cause of miscarriage.

“Miscarriage can come when you fight with someone and you are hit in the wrong place.” (*FGD10, P9*)

“One can miscarry if you are shocked by bad news.” (*FGD10, P4*)

Hereditary transmission (referred to as “hereditary” or “inherited trait”) was a prominent sub-theme for congenital anomalies. Many women reported that the mother of a child with a congenital anomaly is often thought to have conceived with a man other than the husband, since the husband did not appear to have, or physically display, the same congenital anomaly. This highlights an underlying theme of mistrust and blaming of mothers for any adverse pregnancy outcome (explored more below under “cultural beliefs”). Incest was also cited as a cause for congenital anomalies and miscarriages. Incest was reported as a reason to kill a baby born with a congenital anomaly by one group.

“Sometimes they would say you conceived with a relative, so most people normally kill them [child with congenital anomaly].” (*FGD6, P7*)

#### 2: Cultural Beliefs

There were many beliefs around the causes of miscarriage and congenital anomalies including extra-marital sex, not respecting traditional ways, and being cursed or possessed. 

A strong theme for causing both miscarriage and congenital anomalies was infidelity, where the woman conceived outside of wedlock. This includes being raped, although this could also be associated with the physical strain and emotional distress caused, if the husband cheated on the wife, and as a consequence to incest.

“I have heard one but I’m not sure about it, that if you spend [a night] with a man other than your husband when pregnant then you can abort.” (*FGD9, P9*)

“For a baby born with a defect, people would think how possible it is especially if the mother has no defect. Someone would think how she can give birth to a child with a defect like having only one eye. She would think that perhaps this might have come as a result of going out with other men.” (*FGD8, P6*)

Women in different groups reported that if their husband either had an extra marital affair or had slept with an inherited wife, then the current wife would be more likely to miscarry or bear a child with a congenital anomaly.

Not conforming to traditional rules was mentioned as a cause of miscarriage and congenital anomalies. Mostly this related to not performing traditional rituals surrounding marriage, becoming pregnant before the husband paid the bride-price, or building a new house while the wife is pregnant. For the latter it was not clear whether this was a consequence of physical exhaustion of the pregnant woman, or the breaking of a specific traditional taboo. Other beliefs that could all lead to adverse pregnancy outcomes included the breaking of the taboo around sharing the cooking place with a grandmother, taking on the responsibilities of elders before reaching this senior position, planting when not the owner of the field, or eating meat from a pregnant cow. Many mentioned that adverse outcomes occurred because the woman or her family broke a taboo that was prohibited by the clan and had been cursed. Being possessed by demons was considered another possible cause of a miscarriage or a congenital anomaly.

“If she did something prohibited in her clan and then she gives birth to a deformed child then the community would say it came as a result of committing a taboo.” (*FGD4, P4*)

“A curse can fall upon you depending on how you live in the home or according to some cultural practices that you are supposed to do.” (*FGD9, P5*)

The above quotation indicates a very strong relationship between the breaking of taboos, against cultural norms, and subsequent adverse pregnancy outcomes. These taboos appear to be deeply rooted within the cultural belief systems, and across all age groups. Although the community under study are largely Christian, and very religious, only a few suggested that adverse pregnancy outcomes happen without explanation or have a spiritual/religious explanation as “God’s plan.”

“There is malformation that comes as a result of God’s creation; you can give birth to a child without knowing that it will have a malformation. So that is the will of God.” (*FGD8, P5*)

### Stigma associated with miscarriages and congenital anomalies

A number of the causes given were associated with blame, either on the mother or her family, such as a woman’s infidelity to her husband, or breaking a traditional taboo. This could lead to distrust between husband, mother-in-law and the wife/mother. Women reported that mothers are thus stigmatised rather than supported after going through the distress of having an adverse pregnancy outcome. Some reported that women who suffered from a miscarriage were ostracised and not allowed to come out of their home until they had been cleansed by a spiritual healer. Other pregnant women were also not able to come into contact with them as this might be passed on and cause them also to miscarry.

“In our place, I normally see when one has miscarriage they are not allowed to go out. They say that you stay in the house until maybe the church members come and clean you that is when you can move out. So the reason why they don’t want people to go out and I don’t know what would happen if you went out.” (*FGD7, p8*)

Neglect of children born with abnormalities was reported by a number of groups. Children with any congenital anomaly/disability were seen as not “useful” to the family and an extra burden, as well as stigmatized. This stigma might imply some taboo had been broken. Many respondents said such children are often hidden in homes or even killed. Only three women mentioned that they would love and provide the extra-care the child needed.

“You would not love such kind of a child [with congenital anomaly]; I would not love the child because I would feel that he will not be of any help to me. And then taking care of him would be hard.” (*FGD9, P5*)

“I would love the child [born with a birth defect/disability] because he/she is mine; I’m the one who gave birth to her/him.” (*FGD9, P1*)

“Sometimes the grandmother speaks a lot and she might say that the child is not of her blood.” (*FGD7, P7*)

### Health-seeking behaviour

Participants reported that women seek care either from a health facility or from a traditional healer such as a TBA, herbalist, spiritual healer or “Jarwecho” (a special healer that deals with skeleton issues and bone fracture). Women also may seek advice from family members such as a grandmother, mother-in-law and/or the husband. Women reported that they wanted to seek care from health facilities so that they could be treated in the event of complications (i.e. when there is excessive bleeding), and also so that they could better understand the potential causes of the adverse outcome. Many women reported they would go to the TBA to get herbs or go to see a spiritual healer who would “pray for them” or cleanse them of the curse. In the case of a miscarriage, it seems women would only seek care if there were perceived complications. Most women would otherwise keep the miscarriage a secret. Many women reported that they, and other women, would not seek care if their baby was born with a congenital anomaly.

*M: “When they miscarry, do they seek for any care?”*

*P7: “They don’t seek for any care when they miscarry.”*

*P1: “Sometimes they go to the hospital.”*

*P7: “Some people would not like others to know if they have miscarried.” FGD8*

“From what I hear, there is nothing one can do about it [child born with malformation] and no one can ever give you a piece of advice on how they can be helped.” (*FGD10, P7*)

The decision about where to seek care was discussed by one participant, who noted that it depended on the cost of care and resources available to the family. 

## Discussion

Using FGD methodology, we explored the community explanatory models for adverse pregnancy outcomes and how these explanatory models might influence health-seeking behaviours. Although various biologically plausible causes of miscarriage and congenital anomalies were known, cultural beliefs seem to play a central role in the community perception of these adverse pregnancy outcomes. Such beliefs can result in stigma and influence health-seeking behaviours, as reported in previous studies on relatively minor but disfiguring congenital anomalies [18]. Stigma seems to be strongly related to the belief that the woman either cheated on her husband or she or her family had broken some traditional taboo. Some of the cultural beliefs around miscarriage and congenital anomalies lead to stigmatisation, with the mother largely held responsible for the cause of the malformation; this may have a negative impact on health-seeking behaviours and disclosure of such pregnancy outcomes. Women who experienced adverse pregnancy outcomes are thus often additionally burdened by negative attitudes and stigmatisation from the community. In particular, mothers of children with congenital anomalies were often blamed for cheating on their husbands or being cursed because they have apparently transgressed some cultural taboos. Similar studies from rural India and Nigeria found that parents of disabled children were socially marginalized because of widely held beliefs that they had broken a taboo [19], and their children were neglected [20]. Improving access to information about the possible causes of such adverse outcomes may help to reduce the stigma and shame that these women undergo and increase access to formal healthcare providers. 

These data suggest that mothers of children with congenital anomalies or disabilities do not know where they can seek help or whether any help is available. This, together with social stigma, may contribute to the neglected care of these children in this setting. Other factors such as perception of treatment effectiveness, satisfaction with health care services, and external barriers (e.g. financial constraints, accessibility of health services) also play an important role in driving health-seeking behaviours. We note that limited disclosure of adverse pregnancy outcomes could also impact on the accuracy of public health surveillance programmes and could lead to an under-estimation of the problem. 

Fertility and successful childbearing remain very important factors for status and recognition of a woman’s worth in society, in many countries, including those in sub-Saharan Africa. Childbearing confers social status and strengthens conjugal relationships, and children are seen as contributors to the daily labour. The presence of children ensures the rights of property and maintains the family lineage, as described more fully by Dyer [21]. Unsuccessful childbearing and adverse pregnancy outcomes could thus be a potential cause or driver of gender-based violence. Gender-based violence is widespread in Kenya, and particularly high in Nyanza Province where 60% of women report having ever experienced emotional, physical, or sexual violence by their husbands [14]. Although this was not reported in our FGDs, we noted that females in the focus groups considered violence and rape to be causes of miscarriage. We also recognise that in some countries, with strict abortion laws and poor access to contraception, and family planning, women may be driven to take medicine perceived locally to induce abortion, but which may be potentially teratogenic. This requires further study. 

It is not clear how the overlap between biomedical knowledge and cultural beliefs is incorporated into society’s perception, whether these beliefs are mutually exclusive, or blend according to different societal pressures. Under the biomedical paradigm, illness and disease are attributable to biologically plausible causes based on scientific theories such as factors with biochemical effects. If understanding of ill health is based on a biomedical paradigm, the individual is more likely to seek medical care from modern rather than traditional sources [22]. On the other hand, cultural beliefs are based on supernatural phenomenon, traditional values or religious beliefs. Within this explanatory model individuals would seek help or care from either a spiritual or traditional healer [22]. However, adverse health outcomes are not always categorised as one or another. For example Hausmann-Muela et al [23], reported how in Tanzania there is a widespread belief that witchcraft can affect biomedical treatment of malaria and impede detection of malaria parasites. Addressing such beliefs, and acknowledging the role of traditional healers and birth attendants, should be part of public health and research programs. Creating collaborative links between traditional and modern medicine, such as by empowering TBAs to refer their clients to health facilities, is critical to increase access to care in developing countries [24]. 

There was an underlying theme of trust among this rural population in western medicine and healthcare providers. Medications were deemed safe when prescribed and when taken at the correct dosage as prescribed, and adverse outcomes were considered to be due to a lack of medical follow up for women who didn’t attend ANC. Similar to other studies, our research findings suggest that women in rural African settings might suffer from a “white-coat complex” where they avoid asking questions to healthcare workers, and assume any prescribed drugs are safe [25,26]. At the same time, however, pregnant women do not always take medicines given at the health facilities, not because they fear the potential teratogenic risk, but because pregnancy predisposes them to nausea. This has important implications for any intervention targeting pregnant women, and consideration should be given to this to ensure compliance, including the use of directly observed therapy where feasible. Furthermore, in a context where healthcare clients do not ask questions and rely on the providers’ judgement, it is particularly important that healthcare providers who see pregnant (or potentially pregnant) women should be familiar with all drugs contraindicated during pregnancy and potential teratogens. Whilst women were aware of the potential risk of using medication during pregnancy, most did not know the potential risk in the first trimester. The few specific drugs that they did mention (such as quinine and chloroquine) are safe for use in all trimesters of pregnancy, exemplifying this population’s lack of knowledge. Others mentioned that antimalarials should be avoided towards the end of pregnancy in the third trimester. This highlights the need for more information, education and communication on the risk of medication used during the first trimester of pregnancy and the importance for women to explicitly disclose their potential pregnancy to healthcare providers. The widespread belief that modern family planning methods increases the risk of having a child with congenital anomalies also urgently needs to be addressed, particularly within the context of a country with a high fertility rate since the 1990s [27]. Family planning interventions that promote healthy spacing of pregnancy are vital for development and it will be important to ensure accurate information is available to offset myths which could negatively impact uptake of family planning programmes. Only about 25% of women in the study area report using a family planning method (unpublished data), compared to 73% nationally [14].

This study has some possible limitations. Despite efforts to provide a non-threatening environment, respondents may have withheld information and some members might have been affected by a social desirability effect. FGD participants could have been inclined to give what they thought would be acceptable answers, and may have feared telling the truth, particularly around local myths and taboos. This is exemplified by the fact that most of the reported themes under cultural beliefs were reported in the third person (i.e. “it is believed that” rather than “I believe”). The FGDs were conducted as formative research for setting up a pregnancy pharmacovigilance study, which may have influenced the moderator and the participant to focus on issues around drug safety. Although one of the coders (S.D.) is the study coordinator for the pharmacovigilance study and has a specific interest in perception of drug safety in pregnancy, the second coder was not associated with the pharmacovigilance study. Many topics were included in the FGDs which limited the depth and details of the information collected. Moreover, the findings reflect the circumstances affecting a particular population, i.e., women in one rural area in western Kenya, and might not be generalisable. Although validation of findings representing the views elicited through 10 FGDs through data triangulation was not possible, investigator triangulation (where two investigators independently coded the data and compared notes) was used to enhance credibility of the emerging sub-themes. This study did not include the perspective of men or healthcare providers who often play an important role influencing healthcare seeking behaviours. Future studies including such groups would contribute additional understanding to the barriers to healthcare seeking for adverse pregnancy outcomes.

## Conclusion

To our knowledge, this is the first qualitative study undertaken in Kenya exploring the perceptions, attitudes and health-seeking behaviours on adverse pregnancy outcomes. Better understanding of these concepts can inform strategies to improve women’s health-seeking behaviour. Development of appropriate information, education and communication and outreach materials should inform women of true causes of adverse outcomes. This will also help to reduce the burden of guilt they feel, the stigmatisation from the community, and provide guidance on caring for women who have adverse pregnancy outcomes and have children with disabilities. To support this, there is a need for greater integration and collaboration between traditional healers and modern medical practitioners, and to ensure culturally acceptable management to better protect the unborn child and expectant mothers. 

## Supporting Information

Table S1 **Main themes and sub-themes used in the thematic analysis for exploring risk perception and attitudes to miscarriage and congenital anomaly.**
(PDF)
