# Supplementary material for: Exploring Risk Perception and Attitudes to Miscarriage and Congenital Anomaly in Rural Western Kenya
Source: PLoS One. 2013 Nov 13;8(11):e80551. doi: 10.1371/journal.pone.0080551 (PMC3827434; doi:10.1371/journal.pone.0080551)
Supplement: Table S1 — Main themes and sub-themes used in the thematic analysis for exploring risk perception and attitudes to miscarriage and congenital anomaly. (PDF) [file pone.0080551.s001.pdf]

Table S1. Main themes and sub-themes used in the thematic analysis for exploring risk perception and attitudes to miscarriage and congenital anomaly

Themes	Sub-themes
Causes	Biomedical
	Illnesses<br>Medications<br>Stress<br>Hereditary
	Cultural
	Not conforming to traditional norms<br>Infidelity<br>Curse
	None
Stigma	Stigmatisation
	Hide child with abnormality<br>Isolate woman who had a miscarriage<br>Blame/Gossip<br>No disclosure
	No stigma
	Accept child with a congenital anomaly<br>Freely disclose adverse pregnancy outcome
Health-seeking behaviours	Health facility
	Traditional
	TBA<br>Spiritual healer<br>Herbal remedies
	Family
	Husband<br>Parents in law<br>Grand-mother
	None
